# A Community Grounded Approach to Addressing COVID-19 Health Disparities and Misinformation in Latinx and Indigenous Mexican Immigrant Communities

**DOI:** 10.1007/s10900-026-01615-5

**Published:** 2026-07-25

**Authors:** Noah Baltrushes, Ashley Trinidad, Evelyn Vázquez, Elena Kozlova, María Pozar, Ann Marie Cheney

**Affiliations:** 1 School of Medicine, University of California Riverside, Riverside, CA USA; 2 Department of Social Medicine, Population, and Public Health, School of Medicine, University of California Riverside, Riverside, CA USA; 3 Deparment of Psychiatry and Neuroscience, School of Medicine, University of California Riverside, Riverside, CA USA; 4 Conchita Servicios de la Comunidad, CA Mecca, USA

**Keywords:** COVID-19, Vaccine hesitancy, Health misinformation, Institutional mistrust, Latinx immigrants, Indigenous Mexican communities, Community-based participatory research, Health equity

## Abstract

The COVID-19 pandemic disproportionately impacted historically marginalized populations, especially low-income Latinx and Indigenous Mexican immigrant communities in the United States. These groups face persistent structural and social obstacles to health, such as limited healthcare access, language barriers, and concern regarding immigration status, that contribute to institutional mistrust, health misinformation, and reduced COVID-19 vaccine uptake, deepening existing public health disparities. This mixed-methods study utilized a community-based participatory research (CBPR) approach to address vaccine hesitancy among Latinx and Indigenous Mexican farmworkers in the Eastern Coachella Valley, a rural desert region in Inland Southern California. A toolkit, featuring a mural, mini-documentary, and community talks, was developed and implemented to promote COVID-19 vaccination. Pretest and posttest surveys were conducted with 94 community members and three focus groups with 39 total participants provided qualitative feedback on the intervention. The intervention reduced belief in COVID-19 misinformation and increased trust in institutions (*p* < 0.001, *p* < 0.001). However, no significant improvements were found in reducing fear and insecurity surrounding vaccination or English-language confidence (*p* = 0.557, *p* = 0.292). Focus group feedback highlighted structural inequalities as contributors to mistrust in healthcare and vaccine hesitancy. Community-based interventions tailored to cultural and linguistic contexts show promise in reducing misinformation and improving institutional trust in marginalized communities. However, deeper structural inequities, such as racism and economic instability, must be addressed to reduce vaccine hesitancy in vulnerable and marginalized communities.

**Supplementary Information:**

The online version contains supplementary material available at https://doi.org/10.1007/s10900-026-01615-5.

## Introduction

In 2019, the World Health Organization identified vaccine hesitancy as one of the top ten global threats. During the COVID-19 pandemic, vaccine hesitancy was a significant driver of disparities in infection, morbidity, and mortality. [1] In the United States (US), although the pandemic affected all populations, its burden fell disproportionately on historically marginalized groups, including Latinx and immigrant populations, who face long-standing structural barriers to healthcare access and public health information. [2–5].

A growing body of research demonstrates that understandings of vaccine hesitancy in these communities needs to contextualize individual-level knowledge or attitudes within broader social and structural conditions shaping healthcare access, including limited English proficiency, non-citizen status, lack of insurance, and experiences of discrimination within healthcare and government systems. [6–9] These conditions limit access to culturally and linguistically relevant health information and healthcare services contributing to institutional mistrust. [6] During the pandemic, such mistrust was further compounded by inconsistent public health messaging and broader sociopolitical dynamics, including anti-immigrant rhetoric and fears related to immigration enforcement. [10, 11 This mistrust has historical roots that extend beyond the pandemic. Latinx and Indigenous communities in the United States carry generational experience of medical exploitation, including non-consensual experimentation, coercive sterilization, and exclusion from healthcare systems, conditions that have produced a justified wariness of government-sponsored health initiatives that cannot be reduced to individual attitudes or knowledge deficits. [6] Trustworthiness, as distinct from trust, refers to the demonstrated behaviors and institutional commitments that make trust rational - and it is the absence of demonstrated trustworthiness, not irrationality, that underlies much of what public health has labeled vaccine hesitancy in these communities.

Limited access to reliable, language concordant health information increased reliance on informal social networks, where health misinformation rapidly circulated. [12, 13 In communities with limited formal education and few Spanish-language or Indigenous-language resources, these dynamics heightened vulnerability to misinformation. [14] Within such communities, mistrust in institutions and government-sponsored programs is shaped by long-standing structural conditions rather than knowledge deficits, highlighting the limitations in public health messaging that frames vaccine hesitancy as a problem of individual attitudes, beliefs, and knowledge gaps. [6, 9, 15].

Building on this body of knowledge, this study integrates the Health Belief Model (HBM) [16, 17 with sociological theories of structural vulnerability. [18, 19 HBM posits that health behaviors are shaped by perceived susceptibility and severity to illness, perceived barriers to and benefits of a recommended behavior, and cues to action. While the HBM operates at the individual level, these perceptions are produced and shaped by broader social and structural contexts. Here, we situate individual-level decision-making within social, economic, and political hierarchies that produce vulnerabilities to structural inequities such as conditions of economic precarity, immigration-related fear, linguistic exclusion, and limited healthcare access, which shape health outcomes among structurally vulnerable individuals and populations, including Latinx and immigrant communities. [18, 19].

We also build on existing evidence highlighting the effectiveness of community-based interventions that leverage trusted local leaders and culturally tailored messaging to address vaccine hesitancy. [20] We use the term ‘community grounded’ to describe an approach that extends beyond CBPR’s principles of shared governance and co-production by embedding research design, implementation, and dissemination within existing community structures and relationships rather than building parallel research infrastructure. This distinction matters for sustainability: community-grounded approaches aim to leave capacity within communities rather than within academic institutions. Arguably, approaches that meaningfully engage community in shared decision-making can shift public health efforts away from traditional, top-down government-centered models, often associated with historical and ongoing harms, toward community-grounded strategies that build trust, counter misinformation, and promote collective wellbeing. [21, 22].

Towards this end, we tested a community-based intervention designed to improve attitudes toward COVID-19-related healthcare services, including testing and vaccination, among low-income Latinx and Indigenous Mexican immigrants in rural farm working communities, a population disproportionately affected by the COVID-19 pandemic. [14, 23 Our study examines how socio-cultural and structural factors shape vaccine hesitancy and evaluates the effectiveness of community-based strategies in reducing mistrust and improving engagement with public health efforts.

We present findings demonstrating how institutional mistrust and structural inequalities shaped engagement with COVID-19 public health measures and how community-based interventions can mitigate these barriers. Study findings contribute to a growing body of evidence that trusted community leaders such as community health workers (CHWs)/promotoras play a critical role in countering misinformation, rebuilding trust in institutions, and strengthening public health responses in marginalized communities. [21, 24, 25].

## Methods

### Study Design and Objectives

This study was part of the Share, Trust, Organize, Partner COVID-19 California Alliance (STOP COVID-19 CA), a National Institutes of Health (NIH) Community Engagement Alliance (CEAL) funded project, that consisted of a state-wide collaborative of 11 academic sites and over 75 community partners in California. The STOP COVID-19 network built the capacity of community academic partnership teams to address COVID-19 related health disparities in underserved populations throughout California. [26] Our community academic partnership focused on engaging rural inland southern California Latinx and Indigenous Mexican immigrant communities in research. We used a community-based participatory research (CBPR) approach to carry out the study, collaborating with community leaders and bilingual (Spanish and English) medical and pre-medical students to address vaccination mistrust. The CBPR framework promotes collaboration with community members, resource sharing, and decision-making. [27, 28].

Community partners contributed to all phases of the research process. In Phase 1, CHWs/promotoras trained in qualitative research methods led data collection, conducting focus groups and interviews with community members in the Eastern Coachella Valley with the support of the academic team. Phase 1 findings were analyzed and interpreted collectively by the community-academic partnership team to identify priority intervention targets. During intervention design, community workgroup members met virtually 12 times to guide content development, ensuring that intervention materials reflected community-identified experiences and needs. In Phase 2, CHWs/promotoras led recruitment, administered pretest and posttest surveys, and facilitated focus groups. CHWs also participated in codebook development and contributed to the analysis and interpretation of qualitative findings. Study findings were disseminated back to the community through community talks, a report produced in partnership with community members, and an event honoring the mural. [29].

**Fig. 1:**
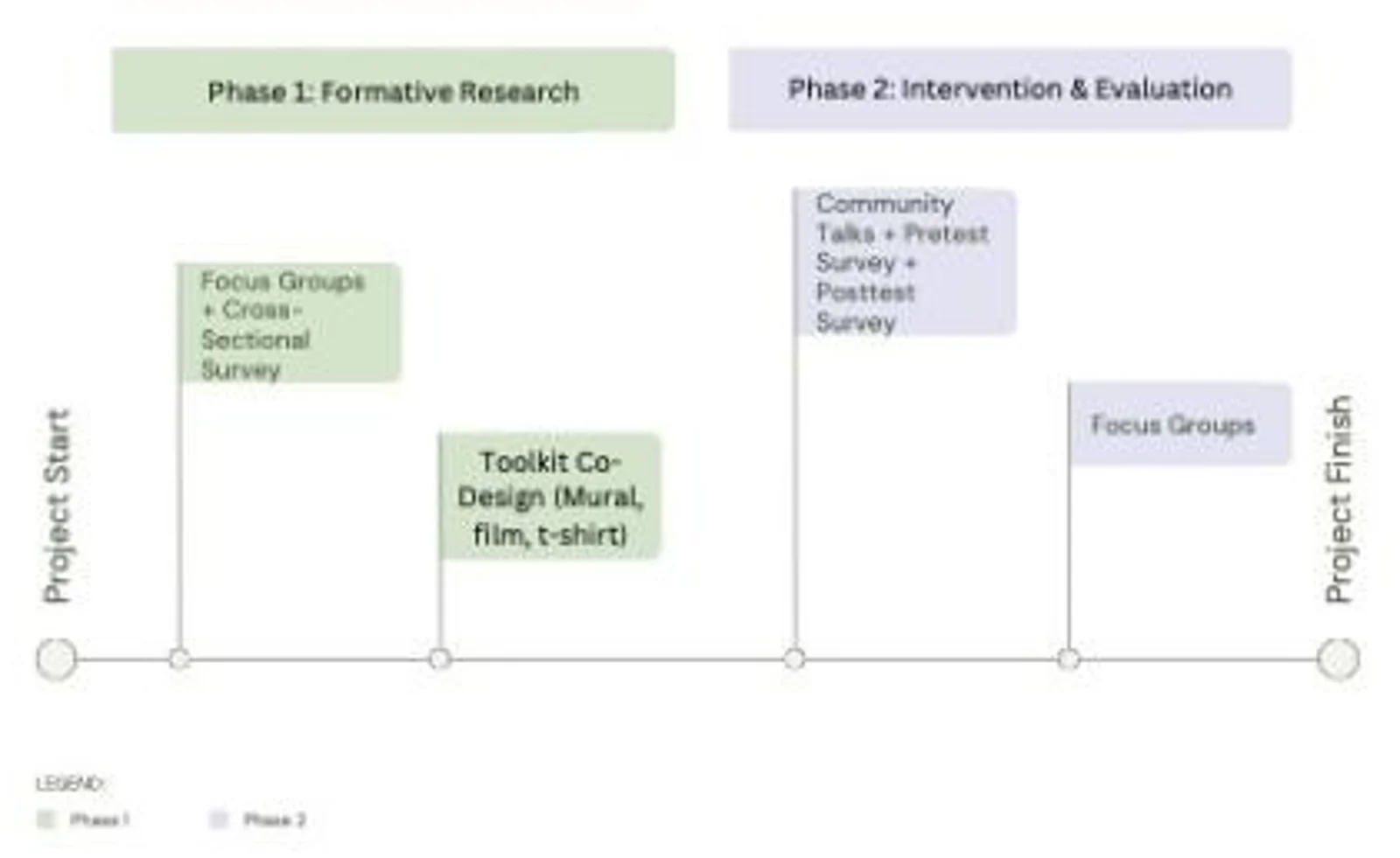
Timeline of project activities by phase

The analysis presented in this article is part of a larger, two-phase mixed-methods study (see Fig. 1). Phase 1 included focus groups and a cross-sectional survey on the socio-cultural and structural barriers to COVID-19 testing and vaccination among low-income Latinx and Indigenous Mexican immigrants in rural communities. As reported elsewhere, fear and mistrust of institutions, along with limited English proficiency in immigrant communities, undermined public health efforts to mitigate virus spread and COVID-19 infections. [6, 14, 23] Using data generated from Phase 1, we developed an intervention consisting of a toolkit with COVID-19 resources to promote the use of COVID-19 public health mitigation efforts, including testing and vaccination. [29] The toolkit comprised a mural titled “Luchando por la Salud de la Comunidad” [“Fighting for the Health of the Community”] and a mini-documentary on COVID-19. In Phase 2, we implemented the intervention and tested its effects on COVID-19 related attitudes and behaviors. This article presents findings from Phase 2: Intervention and Evaluation.

### Setting

The research was conducted in the rural desert region of Inland Southern California, specifically the Eastern Coachella Valley (ECV) in Riverside County. Over 2 million Latinos live in Riverside County, a population that experiences health inequities related to low incomes, limited access to formal education, language barriers, and immigration status. [30, 31] The COVID-19 pandemic exacerbated these pre-pandemic conditions contributing to increased risk for COVID-19 infection and related health disparities. [32].

This phenomenon was especially apparent in the Coachella Valley, a region marked by stark racial and ethnic inequalities. The affluent western valley city of Palm Springs, which is 75% white, contrasts with rural agricultural areas such as Mecca, North Shore, Oasis, and Thermal in the eastern valley, which are home to about 88,000 residents, 97% of whom identify as Hispanic/Latino. In these communities, an estimated 30–50% of residents live below the federal poverty line. [33] These factors predisposed the ECV to viral spread with reports indicating a COVID-19 infection rate 5 times that of other Coachella Valley Communities. [34].

### Intervention

We used the Analysis, Design, Development, Implementation, Evaluation (ADDIE) model to engage members of Latinx and Indigenous Mexican communities and healthcare professionals in the analysis of Phase 1 findings and the initial design and development of the intervention. [35] Following the ADDIE model, we convened a workgroup to engage community members with lived experience of COVID-19 infection.

A total of 10 workgroup members met virtually 12 times to offer their input and feedback on the draft intervention. The workgroup was comprised of community members with lived experiences of COVID-19 in the Eastern Coachella Valley, CHWs/promotoras, a videographer and a community artist convened by the academic-community partnership team. After the workgroup finalized their recommendations for the intervention, we followed common intervention mapping (IM) steps to identify the desired behavior changes and health outcomes, select intervention strategies, structure the delivery and implementation of the intervention, and evaluate its effectiveness on COVID-19-related health behaviors. [36] Community insights, such as distrust of the government, widespread vaccine misinformation, and limited English proficiency, emerged as key factors influencing healthcare decisions and contributed to some individuals choosing to forgo COVID-19 testing and vaccination. These insights directly informed the intervention’s design and development.

The community-determined intervention is a 60-minute interactive virtual talk on the lived experiences of survivors of COVID-19, and lives lost to COVID-19 in rural Latinx and Indigenous Mexican immigrant communities. The talk begins with the narration of a mural, painted by a community artist, depicting pre-pandemic conditions, the wake of the pandemic in rural agricultural communities, and the role of public health efforts, especially those of CHWs/promotoras, in mitigating the effects of the pandemic in Latinx communities (see Supplementary Fig. 2). Central to the mural’s design is the Tree of Life (Árbol de la Vida), a traditional Mexican folk art form with roots in both pre-Columbian Indigenous and colonial-era Catholic traditions, representing the cycle of life, death, and generational continuity. The workgroup selected this symbol for its established resonance within the Purépecha and broader Latinx community and for its capacity to hold collective grief and resilience within a single image. This is followed by a 10-minute documentary featuring testimonials by those who had COVID-19 and sharing of COVID-19 public health mitigation strategies (testing and vaccination) by a Latinx Spanish-speaking physician and CHWs/promotoras. The final component of the intervention was a t-shirt imprinted with the slogan “Luchando Para la Salud de Nuestra Comunidad” [Fighting For the Health of Our Community]. Participants were asked to wear the t-shirt following their participation in the intervention. CHWs/promotoras delivered the intervention, which was designed for delivery over the internet via Zoom or in-person in an outdoor community setting.

### Recruitment and Eligibility

Eligibility criteria included: age 18 or older, residence in ECV, self-identified Latinx or Indigenous identity, and primary language of Spanish or Purépecha. Convenience and purposive (nonrandom) sampling were used to recruit participants into the study. Community health workers (CHWs)/promotoras distributed the study flyer, including contact information and a QR code linking to the eligibility screener, through their community networks and provided participants with support to navigate virtual meeting technology (Zoom).

### Data Collection

Data collection was carried out in February and March 2021. Trained CHWs/promotoras administered pretest and posttest surveys via Zoom, phone, or in-person (outdoors with masks). Pretest surveys were collected within the week prior to the start of the intervention and posttest surveys within three days of the close of the intervention. Both surveys took approximately 20–25 min to complete. Focus groups were conducted with participants post intervention.

*Pretest and posttest surveys*. Participant characteristics were collected at pretest, including age, gender, racial/ethnic heritage, country of origin, maternal language, health insurance coverage, marital status, educational attainment, employment status, COVID-19 history and vaccination status. Changes in attitudes, beliefs, and knowledge related to COVID-19 and vaccination were evaluated by comparing pretest and posttest survey scores using the following scales: misinformation, mistrust in institutions, fear of COVID-19 vaccination and testing, and limited English proficiency using a 3-point Likert scale where 1 = agree, 2 = neither agree or disagree and 3 = do not agree (see Supplementary Table 1 for Scales). The scales were developed specifically for this study and Phase 1 data, including focus group and survey data, informed item development for the subscales. Subject matter experts, including academic and community members involved in both COVID-19 public health efforts and healthcare services delivery for the focal population, reviewed the subscales (misinformation, mistrust, fear, and limited English proficiency) and questions to measure each item per subscale, and provided feedback that was incorporated into the final scale (see Supplementary File for Pretest and Posttest Survey Questions). Cronbach’s alpha coefficients were calculated for pretest and posttest scales to assess the internal consistency of each scale determining the extent to which items were correlated representing an underlying construct. A value of α ≥ 0.70 as acceptable, α ≥ 0.80 as good, and 0.90 and above as excellent internal reliability.

*Focus groups.* To assess participant perceptions of the intervention and explore structural and experiential factors shaping COVID-19 vaccine decision-making, three focus groups with 39 participants (10–14 per group) were conducted following the community talks (research indicates that 80% of themes emerge within two to three focus groups, and 90% within three to six). [37] Community health workers and medical and pre-medical students, all of whom received training in qualitative data collection and analysis, conducted the focus groups. A semi-structured interview guide with open-ended questions was used to elicit information on the following topics: (see Supplementary File for the Semi-structured Interview Guide). In addition to facilitating focus groups, students assisted with scheduling meetings, coordinating logistics, and supporting CHWs/promotoras throughout the intervention process.

A semi-structured interview guide with open-ended questions was used to elicit information on participants’ beliefs and attitudes about the virus, its transmission, and factors affecting trust between the community and health institutions. Focus groups aimed to capture feedback on the toolkits and assess participants’ likelihood of sharing the information or engaging others in their networks. Data from these focus groups were analyzed to both understand barriers to vaccination and community perceptions of the intervention’s impact.

### Data Analysis

Focus groups were conducted via Zoom, audio-recorded, transcribed, and analyzed using template and matrix analysis, a rapid qualitative method. [38–40] CHWs and students, trained in this approach, led the analysis with expert support. Data were systematically reviewed and organized into templates based on key topics from the interview guide: perceptions of the mural, the mini-documentary, and the broader STOP COVID-19 intervention. A matrix was then developed to synthesize findings across the three focus groups, facilitating the identification of recurring themes and patterns. This method enabled the team to focus on participants’ perspectives on COVID-19 mitigation strategies and the role of community-based interventions in addressing misinformation and mistrust.

Statistical analysis was performed using IBM SPSS Statistics (version 28). The effects of the intervention on changing attitudes and beliefs around COVID-19 testing and vaccination were assessed by comparing standardized mean differences in pretest and posttest scores using Paired-Samples t-tests. Statistical significance was determined using a two-tailed alpha level of 0.05. Participants were excluded from item analyses if they did not provide a pre- and post-test response to ensure valid pairing.

## Results

### Participant Characteristics

The study included 94 participants, primarily female (80.85%) and predominantly Hispanic or Latino (94.57%) with 88.30% speaking Spanish as their primary language. Over half (57.78%) were employed, with 53.33% working as agricultural laborers. Nearly half (50.56%) were uninsured, and 49.37% reported a positive COVID-19 test (see Table 1 for full demographic characteristics and vaccine history).

**Table 1:** Participants Characteristics and COVID-19 Vaccine History

Value, *N* = 94	*n* (%)
**Gender**	
Male	18 (19.15)
Female	76 (80.85)
**Age**	
18 to 29	19 (20.21)
30 to 39	33 (35.10)
40 to 49	16 (17.02)
50 to 59	16 (17.02)
60 or older	10 (10.64)
**Ethnicity**	
Hispanic or Latino	87 (94.57)
Indigenous Latin American	5 (5.32)
**Birth Place**	
Latin America	83 (89.25)
United States	10 (10.75)
**Language**	
Spanish	83 (88.30)
English	9 (9.57)
English/Spanish	1 (1.06)
Purépecha	1 (1.06)
**Education Level**	
Did not complete elementary school	19 (21.11)
Completed elementary school	16 (17.78)
Completed high school or obtained a high school diploma	19 (21.11)
Completed some community college or associate’s degree	30 (33.33)
Graduated from a university (4 years)	5 (5.56)
Completed postgraduate education (or advanced degree)	1 (1.11)
**Employment**	
Housewife	14 (15.56)
Unemployed	18 (20.00)
Retired	2 (2.22)
Disabled	3 (3.33)
Employed	52 (57.78)
Other	1 (1.11)
**Agricultural Laborer (yes)**	48 (53.33)
Migrant worker	6 (8.82)
**Healthcare Insurance**	
No	45 (50.56)
Yes	44 (49.43)
**Coronavirus Test**	
No, never had a test	9 (10.00)
Yes, I had a test	81 (90.00)
**Positive Covid**	
No, I did not test positive for COVID-19	41 (51.90)
Yes, I tested positive for COVID-19	38 (49.37)
**Vaccinated**	
No	6 (6.52)
Yes	86 (93.48)
**Vaccine Type**	
Johnson and Johnson	11 (13.92)
Moderna	24 (30.38)
Pfizer	44 (55.70)
**First Dose**	
Yes	69 (100.00)
**Second Dose**	
Yes	61 (100.00)
**Booster (yes)**	
Yes	35 (83.33)

### Quantitative Findings

Pretest and posttest survey results from 85 to 92 participants revealed statistically significant improvements in addressing key barriers to COVID-19 healthcare service utilization. The mean misinformation scale scores improved significantly from 2.3232 (SD = 0.46270) to 2.4718 (SD = 0.45529), indicating reduced acceptance of misinformation (t = −3.308, *p* < 0.001). The mean mistrust in institutions scale scores also significantly improved from 2.5009 (SD = 0.54181) to 2.6721 (SD = 0.42352) (t = −3.472, *p* < 0.001). These results provide preliminary evidence that the intervention was associated with reductions in misinformation acceptance and improvements in institutional trust. Effect sizes were small to moderate (misinformation: Cohen’s d = 0.32; mistrust: Cohen’s d = 0.35), consistent with what might be expected from a brief, single-exposure community intervention. Given the absence of a control group, these changes cannot be attributed solely to the intervention, and findings should be interpreted as preliminary and hypothesis-generating (see Table 2).

**Table 2:** Factors underlying COVID-19-related healthcare decision-making

Construct	Cronbach Alpha (pretest and posttest)	Pretest mean (SD)	Posttest mean (SD)	t
Misinformation (*n* = 91)	0.706 and 0.770	2.3232 (0.46270)	2.4718 (0.45529)	−3.308 *p* < 0.001
Mistrust (*n* = 92)	0.838 and 0.793	2.5009 (0.54181)	2.6721 (0.42352)	−3.472 *p* < 0.001
Fear (*n* = 92)	0.867 and 0.774	2.8417 (0.36803)	2.8650 (0.28276)	− 0.590 *p* = 0.557
Limited English proficiency (*n* = 85)	0.859 and 0.833	2.9196 (0.28214)	2.8824 (0.30027)	1.060 *p* = 0.292

**Fig. 2:**
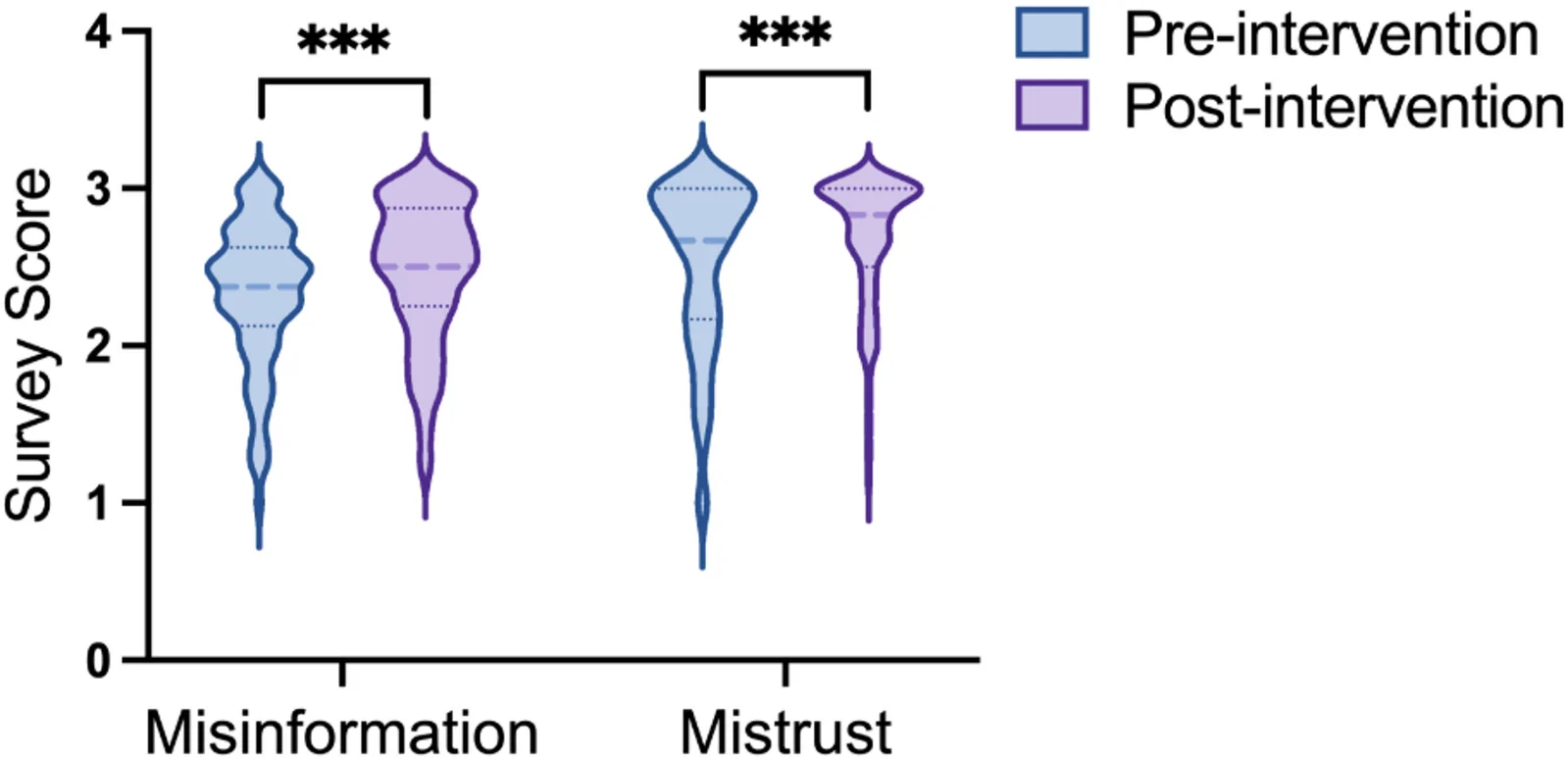
Pretest and posttest intervention results for misinformation and mistrust. *indicates significant vs. pre-intervention using two-tailed paired-samples t-test (***p < 0.001). Violin plots depict median and interquartile range

As shown in Fig. 2, survey responses on misinformation and mistrust scales showed significant increases post-intervention, indicating reduced misinformation and greater trust in healthcare services. However, fear surrounding COVID-19 vaccination and testing did not show a statistically significant change (mean difference = 0.0233, *p* = 0.557). Additionally, there were no statistically significant differences in the proportion of participants with limited English proficiency (mean difference = −0.0372, *p* = 0.292) between pretest and posttest, suggesting that persistent immigration status insecurity and linguistic barriers may have influenced these outcomes.

### Qualitative Findings

Table 3 provides key themes organized by three analytic categories: Symbolism of COVID-19 experiences, memorialization of the COVID-19 pandemic, and community-level impact, and includes participant quotes to provide evidence of the summary descriptions below.

**Table 3:** Perceptions of the COVID-19 Toolkit/Intervention and its influence on COVID-19 decision making

Analytic category and Theme	Participant quotes
**Symbolism of COVID-19 Experience**	
*Struggle and Loss*	
	“I remember the name of [priest]. He seemed to me to be such a good person, so wholehearted… he wanted to do everything. [H]e left a great void… His entire community continues to miss him and it’s a great pain… and unfortunately, we can no longer turn back time, right? *~ Participant in focus group*
	“Personally, I think it’s a very nice detail that they took into account the people who lost their lives.” *~ Participant in focus group*
	“I saw, when my daughter and my wife got the virus, sometimes relatives brought us things, food and left them outside. They looked at us oddly. You feel sad when people look at you like that, you feel ugly, sad. *~Participant in focus group*
*Resilience and Hope*	
	“I really liked the tree, the whole mural, because I think it raises awareness for the community that the vaccine is very important and that it is very important to move forward. Let’s not let our guard down, because we have a future ahead of us that is much further ahead than what has already happened, which has left us with a great pain… in our hearts.” *~ Participant focus group*
	“That [the mural] makes us understand that we have to live life in the present because we don’t know if there will be a future. So, we have to live to the fullest. That [the mural] represents us or gives us a lot of meaning for the whole community, which is that we have a very short life and we don’t know when we will leave.” *~ Participant in focus group*
	“I think it [the intervention] will help us reduce fear a little. As I said, what I liked about the documentary and the shirt is that the shirt says ‘Fighting for the community.’ It means that we are fighting, helping each other and fighting to continue taking care of ourselves and protecting ourselves against the disease, COVID.” *~Participant in focus group*
*Homage to Lives Lost*	
	“Well, each little leaf belongs to a loved one who has passed away. This pandemic is leaving history… and it will leave history for life. It is history and we all know that we have lost many people.” *~ Participant focus group*
	“Remembering the people who really did something in this community is very nice and very valuable, we will never forget it.” *~ Participant focus group*
	“When asked the question about the mural, if there were loved ones on it, the names of our loved ones. I actually wanted to answer, but it’s very sad to know that there were. Unfortunately, on that mural I have three of the people who have been very, very dear to me. For example.. the name of my mother-in-law, a person who, thank God, was very healthy. She did have bone pain… but she didn’t suffer from any illness. It was pain due to age. She didn’t suffer from any illness, and she got COVID. Unfortunately, COVID took her away.” *~ Participant focus group*
*COVID-19 Mitigation Strategies*	
	“Because we are doing a kind of dissemination of what the mural is… we were looking at what is the vaccination, wearing masks, being careful not to be so close together and not making visits, hearing about places that are very, very full of people.” *~ Participant focus group*
	“I think it is super important to be able to share it [toolkit] with other families who have not yet been vaccinated and for them to think it is necessary to get vaccinated, because we have already gone through several stages of distress since COVID-19 and the strains are becoming stronger…. And still, many people do not believe that this is true. So maybe by presenting them with the evidence, the videos of other families who have already gone through it.” *~ Participant in focus group*
	“We have all gotten sick from COVID at home. That is our fear because, although they say that the number of infected people has decreased considerably, I say that we should still not stop wearing face masks. But many people no longer do. I can already see that people no longer use them. That is why I don’t know what will happen because I know many people who have died from COVID.” *~ Participant in focus group*
**Memorialization of the COVID-19 Pandemic**	
*Lessons for Future Health Crises*	
	“I think that is the idea [of the mural and documentary] that will make people aware and take precautions, although unfortunately not everyone will do it. But those of us who are aware will do so. By setting an example, I also think that it would be a way of raising awareness and taking precautions.” *~ Participant in focus group*
	“Fear is latent…. We have lived with fear, we have also looked at the hospitals, we cannot say: ‘I am not going to take care of myself anymore.’ Because, we do not know. Prevention is better than getting infected.” *~ Participant in focus group*
	“It [the mural] creates a conversation topic that encourages prevention and safety within the community. Hopefully it will alert people to continue taking the proper precautions.” *~ Participant in focus group*
*Historicization of the Pandemic*	
	“I think it’s [the mural] a reflection of what was happening, is happening and what happened. It’s what will be left behind. This is something that people who have not been vaccinated, so that this mural is indicating everything that happened for not getting vaccinated, those who were vaccinated, what did not happen and what happened. That’s what I like and I also see, because there was a lot of connection with the people who were really helping to vaccinate, to help, right?” ~ *Participant in focus group*
	“I think that putting it [the mural] there was something very significant and maybe for us it is something new [the mural] that we had not seen. But for our children, it [the mural] is something normal because they began to see how the pandemic was addressed. By having this mural, they ask questions. For example, when looking at the mural, they can ask us what does this mean? So, the mural also helps teach children what the pandemic was about. Not just for the children to hear about it, but the true meaning and why it is important for them to get vaccinated at an early age as well.” *~ Participant in focus group*
	“I think because they [the leaves on the tree of life] are the loved ones we have lost. Seeing them represented in an artistic, permanent way… I believe it’s something very beautiful, symbolic, something historic as well for the region, for the city.” ~ *Participant in focus group*
**Community-Level Impact**	
*Mitigate Collective Fear*	
	“They [community members] are still afraid of getting the vaccine because they say they are going to turn into zombies. That’s why they don’t want to get the vaccine…. That’s why I say that the mural.. it’s explaining to you exactly that you have to continue …to wear your mask. You have to get the vaccine. Even I was afraid of getting the vaccine, but not because I was going to turn into a zombie, but because I’m afraid of vaccines.” *~ Participant in focus group*
	“As I said, I think that fear is still there, even though things are calmer now. But I think that it is also a way of raising awareness for people, for those who are not vaccinated and especially for people who have been affected.” ~ *Participant in focus group*
	“Many people haven’t gotten it [the COVID-19 vaccine] purely out of fear. Because people tend to say negative things [about the vaccine]… It’s good for people to hear that the vaccine is good so they can understand more about it.” *~ Participant in focus group*
*Vaccine Access*	
	“.. it [the mural] is a way of saying that the vaccine is safe, that many people came out of the darkness into the light. Why? Because thanks to the vaccine… [before the vaccine] we couldn’t go anywhere, we were just locked up. But as soon as the vaccine started, the infections began to decrease. I say that it [the mural] is a way of encouraging people to get vaccinated.” *~Participant in focus group*
	“There were people who thought they were going to be asked for the name of their Medi-Cal [insurance] or something like that. [T]hey tended to be those people who didn’t have Medi-Cal, they didn’t have insurance… In the end they [uninsured persons] were informed that they [healthcare professionals] wouldn’t ask if they didn’t have [insurance]. And, they went with the confidence of being able to get vaccinated.” ~Participant in focus group
	“Many people were asking if their personal information was going to be shared with people in immigration.. I think that helped them [uninsured persons] decide to get the vaccine because they no longer felt at risk and afraid of their information being shared.” *~ Participant in focus group*
*Effect of intervention toolkit on public discourse*	
	“The mural looks really nice. It does motivate people. If you look at it, the mural is motivating… and it is very well done. And there are the perfect t-shirts for, for example, when you go to mass and you put it on and people look at you from behind…. And they ask you: ‘Who gave it to you? Did you buy it?” *~ Participant in focus group*
	“When you go to the swap meet, you put it [t-shirt] on and people look at you, intrigued by the nice shirt … it’s [the mural image] drawn really nice, the mural looks good…. I go to car shows and I put it on … to explain to them …. *~ Participant in focus group*
	“I’m walking around the swap meet. I came with my husband and that’s why sometimes the voices would stop a little. But yes, I think it’s [the t-shirt] a form of promotion. More than one person is curious, what does this symbol mean?” *~ Participant in focus group*

#### Symbolism of COVID-19 Experiences

The intervention, specifically the artwork on the mural, resonated with participants, who noted the darker tones illustrated the struggles and losses experienced during the pandemic, while the brighter tones conveyed resilience and hope. Each leaf on the mural’s tree symbolized lives lost, reminding participants of the pandemic’s continued impact and historical significance. The intervention promoted awareness and a renewed commitment to COVID-19 safety measures, including mask-wearing, social distancing, and vaccination. However, some participants indicated that broad adoption of these precautions remained a challenge.

#### Memorialization of the COVID-19 Pandemic

The locally specific imagery of the mural reminded participants of the challenges faced together. Participants saw the mural as a place to honor loved ones who had passed away, with one participant saying, “This pandemic is leaving history… and it will leave history for life.” The “tree of life” depiction, in particular, resonated with those who had lost people to COVID, helping them to process their grief and providing a visible reminder of the pandemic’s toll. Participants noted that the mural could serve as an educational tool, helping to contextualize the pandemic’s impact and the importance of public health practices.

#### Community-Level Impact

The mural and branded t-shirts directly addressed public fears and promoted vaccine access. The documentary and mural helped confront persistent fears about vaccination (e.g., myths about severe side effects) by providing information and representing the work done by community health workers during the pandemic. For many, the mural reinforced the importance of safety behaviors and encouraged them to view vaccination as a shared responsibility. Participants said this was supported by the documentary, which they felt highlighted the dangers of not being vaccinated. Additionally, the intervention addressed worries about immigration status and lack of insurance, which allowed more individuals to feel secure in seeking vaccination. The t-shirts extended this effect by sparking curiosity and conversation, engaging a broader audience.

These quantitative and qualitative findings can be understood retrospectively through the lens of the Health Belief Model constructs. The intervention addressed perceived susceptibility and severity through survivor testimonials and the mural’s depiction of community loss; perceived barriers and benefits through direct engagement with structural concerns including immigration fear and insurance access; and cues to action through the mural, documentary, and t-shirt as prompts extending reach into community social networks. Table 4 summarizes how each HBM construct corresponds to a specific intervention component and its target outcome.

**Table 4:** Health Belief Model constructs and their relationship to intervention components

HBM Construct	Definition as applied here	Intervention component	Target outcome
Perceived susceptibility	Belief that one is at personal risk for COVID-19	Documentary: survivor testimonials from community members	Increased recognition of personal risk
Perceived severity	Belief that COVID-19 poses serious health consequences	Mural: depiction of community loss and pandemic toll	Increased awareness of pandemic severity
Perceived barriers	Structural and attitudinal obstacles to vaccination	Community talks: addressed immigration fears, insurance access, and misinformation	Reduced mistrust and misinformation
Perceived benefits	Belief that vaccination reduces risk of serious illness	Documentary: CHW and physician messaging on vaccine safety and efficacy	Increased confidence in vaccination
Cues to action	External prompts that motivate health behavior	T-shirt, mural, and community talks extending reach into social networks	Extended community reach and conversation about vaccination
Self-efficacy	Confidence in one’s ability to act on health recommendations	CHW/promotora delivery model: trusted messengers from within the community	Improved engagement with public health services

## Discussion

Three key findings emerged from this study: the value of community-grounded CBPR strategies in addressing health inequities during the COVID-19 pandemic, the preliminary evidence supporting a culturally tailored toolkit in reducing misinformation, and the promise of community-based approaches in building institutional trust within low-income immigrant communities. These findings suggest that shifting public health efforts away from top-down, government-centered models toward community-grounded strategies can more effectively engage structurally vulnerable populations.

The intervention significantly decreased acceptance of misinformation and increased trust in institutions. These results reinforce the established value of CBPR in public health. [27, 28, Collaborating with CHWs/promotoras and community leaders was pivotal in a context where traditional efforts to combat misinformation and promote trust often failed. [12] Structural barriers, including delayed and insufficient Spanish-language messaging, [23] historic institutional mistrust, [18] limited digital access, and literacy challenges [14, 32], hindered centralized public health initiatives. By employing a CBPR approach with shared leadership and community-based design, the intervention succeeded in mitigating misinformation and building trust to address structural barriers shaping individual decisions, attitudes, and behaviors, highlighting the importance of culturally tailored, community-based solutions in mitigating health inequities. [6, 27].

Misinformation such as conspiracy theories and beliefs that the vaccine was a ploy for the US government to insert a chip in, or chemically sterilize, the US populace was common during the pandemic. [41, 42] For Latinx immigrants, socio-economic status, racial-ethnic background, limited English proficiency, and lack of access to public health information and healthcare services necessitated the formation of informal information sharing networks and alternative health-seeking practices, [43, 44] increasing susceptibility to misinformation. [14, 33] Although a 2022 meta-analysis showed only marginally effective misinformation interventions overall in the context of COVID, [45] there was significant heterogeneity in the results. The strategies most strongly correlated with success (high levels of participant engagement in public health and text-only formats) were of limited use in this community due to the lack of high quality and up to date Spanish-language resources and low levels of English literacy and formal education. [14].

In our research, participants valued the inclusion of culturally relevant symbols and narratives in the intervention’s materials, including the mural and mini-documentary, which closed the gap between public health messaging and the community’s lived experiences during the pandemic. The incorporation of community members’ voices and stories contributed to this, resonating with participants’ own experiences, which in turn fostered engagement and trust, leading to a significant decrease in misinformation, according to survey responses.

Another key finding was the effectiveness of the intervention in building trust in US institutions, specifically the government. Members of this community live in the shadow of the historical traumas of colonialism and have little trust in power structures, of which they have consistently found themselves at the bottom over hundreds of years. [6] The mural, in particular, with its celebration of CHWs and emphasis on vaccination as the bridge from COVID to post-COVID conditions, increased confidence in public health initiatives by reframing the narrative as one of collective struggle and triumph, rather than dependency and saviorism. [6] This reframing reflects a broader principle: that community-grounded approaches, rooted in shared decision-making and trusted relationships, can succeed where government-sponsored top-down campaigns have historically failed in structurally vulnerable communities. [18, 20].

Although there were significant and encouraging changes in misinformation and trust in institutions, the intervention did not have a significant effect on fear and insecurity around vaccination or language-specific healthcare navigation confidence. Viewed through the HBM, the significant changes in misinformation and mistrust suggest the intervention may have addressed perceived susceptibility to and severity of infection. Consistent with scholarship on structural determinants of vaccine hesitancy in Latinx communities, [7, 9] the non-significant changes in fear and language confidence may reflect perceived barriers that are structural in origin and not amenable to a single short-term intervention. While strategies like motivational interviewing, prebunking (preemptively correcting misconceptions before misinformation spreads), and social media campaigns have shown promise in reducing vaccine hesitancy and improving healthcare navigation, [46, 47] these interventions were typically longer-term and implemented in demographically distinct settings. Nevertheless, their success highlights opportunities for enhancing our own intervention, particularly by extending its duration, incorporating digital components, and maintaining the CBPR framework tailored to rural Latinx communities.

### Limitations

This study has several limitations. First, the relatively small sample size and focus on a small and specific geographic area may limit the generalizability of the findings to other Latinx communities in dissimilar areas and circumstances. Second, the study’s reliance on self-reported data in the form of surveys and focus groups, although helpful in gathering personal perspectives, could leave responses vulnerable to human error such as recall and social desirability biases. Third, the study, while addressing misinformation and trust in institutions, was not broad enough in scope to address other major contributors to vaccine hesitancy such as structural inequalities and economic instability. Future research would benefit from investigation of a larger number of factors contributing to vaccine hesitancy across a more diverse population over a broader geographic area.

Fourth, while the HBM provides a useful lens for understanding individual-level outcomes in this study, it does not account for the structural and social conditions, including immigration-related fear, linguistic isolation, and economic precarity, that shape those determinants. The lack of significant change may reflect barriers that are not well captured by individual-level behavior analysis. Future research would benefit from integrating a structural or critical framework alongside the HBM to better account for these multilevel conditions. Fifth, the outcome measures assessed behavioral intentions regarding COVID-19 vaccination and public health engagement. Behavioral intentions are associated with but not synonymous with behavior change, and positive shifts in intention cannot be assumed to translate to actual vaccination uptake. Future research would benefit from a post-intervention follow-up to measure vaccine behavior directly, which would more fully substantiate the toolkit as an effective vaccine promotion intervention.

Sixth, the absence of a control or comparison group is a central limitation of this evaluation design. Without a comparator, it is not possible to rule out alternative explanations for observed pre-post changes, including regression to the mean, contemporaneous public health campaigns, or natural shifts in community attitudes over the data collection period. Without a comparator, observed pre-post changes should be interpreted with caution, as alternative explanations including regression to the mean, contemporaneous public health campaigns, or natural shifts in community attitudes cannot be ruled out. Additionally, community-based recruitment through existing networks may have overrepresented individuals already engaged with public health efforts. The structural conditions documented in this literature, including immigration-related fear and institutional mistrust, that contribute to vaccine hesitancy in this population are the same conditions that limit research participation, meaning those most in need of the intervention were least likely to be enrolled. [6].

## Conclusion

This study contributes preliminary evidence that community-grounded, culturally and linguistically tailored interventions show promise in reducing misinformation and building institutional trust among Latinx and Indigenous Mexican immigrant populations facing distinct socio-cultural and structural barriers to healthcare. The intervention’s limited effect on fear and language-confidence outcomes points to the limitations of short-term approaches and calls attention to the need for structural-level interventions to change behaviors shaped by immigration-related fear and linguistic isolation, which are structural in origin and require structural responses. [6, 14] Culturally tailored, community-based interventions are necessary alongside broader systemic changes. In places like the Eastern Coachella Valley equitable outcomes ultimately depend on shifts in policy that address the upstream conditions shaping healthcare access. [15, 18, 19] The present study offers a replicable model for community-academic partnerships working under similar constraints [20, 48], and underscores the value of partnership and centering community knowledge at every stage of intervention design and delivery. [27] These findings have direct implications for future public health responses: investing in community-grounded infrastructure, including CHW networks and community-academic partnerships, rather than relying on centralized government messaging, offers a more equitable and effective path to health equity in structurally vulnerable communities. [21, 24, 25].

## Supplementary Information

Below is the link to the electronic supplementary material.

Supplementary Material 1

Supplementary Material 2

Supplementary Material 3

Supplementary Material 3

## Data Availability

The datasets used and/or analyzed during the current study are available from the corresponding author on reasonable request.
