# Supplementary material for: A Community Grounded Approach to Addressing COVID-19 Health Disparities and Misinformation in Latinx and Indigenous Mexican Immigrant Communities
Source: J Community Health. 2026 Jul 25;51(6):991–1004. doi: 10.1007/s10900-026-01615-5 (PMC13748008; doi:10.1007/s10900-026-01615-5)
Supplement: Supplementary file 1 [file 10900_2026_1615_MOESM1_ESM.docx]

3

**Figure 2**. STOP COVID-19 Mural

This is the mural we used in the intervention. As you transition to the right the sky lightens and community health workers (promotores) are present against the backdrop of a mobile unit and tree of life. The promotores represent the connection of healthcare systems to the community; they became the face of COVID-19 testing and vaccination during the height of the pandemic. They initially connected vulnerable community members to testing services and continued to engage communities in vaccination services.
