# Supplementary material for: A Community Grounded Approach to Addressing COVID-19 Health Disparities and Misinformation in Latinx and Indigenous Mexican Immigrant Communities
Source: J Community Health. 2026 Jul 25;51(6):991–1004. doi: 10.1007/s10900-026-01615-5 (PMC13748008; doi:10.1007/s10900-026-01615-5)
Supplement: Supplementary file 2 [file 10900_2026_1615_MOESM2_ESM.docx]

Supplementary File: Semi-structured Interview Guide

Introduction: Thank you for taking the time to meet with me today. We asked you to do this interview so we can get a better understanding of use of behavioral healthcare services this includes mental health and substance use services in your community. First, we’ll ask you to talk about the free clinic and then about the kinds of behavioral healthcare services in your community. We will also ask you to share your thoughts about some challenges to getting care for mental health and substance use and what would make it easier, especially during the COVID-19 pandemic. Remember there are no right or wrong answers. We would like to record this interview. While audio recording is not required for participation, it is preferred. If participants do not want to be audio recorded, notes will be taken to document information shared. Do we have your permission to record the interview?

Questions:

1. Can you tell me about the kinds of stress and trauma that people in your community have experienced during the COVID-19 pandemic?
   a. Probe: Would you like to share with me any example of stress or trauma that you or someone in your family has experienced?
2. Now let’s talk about your visit to the clinic. Can you tell me about why you decided to go to the Coachella Valley Free Clinic? What brought you to the clinic?
   a. Probe:
      i. How did you hear about the clinic?
      ii. What did you learn about our mental health services?
      iii. How did you get to the clinic? (car, bus, walked)
      iv. Did you experience any challenges to getting to the clinic?
3. What did you think of your visit with the mental healthcare professional?
   a. Probe:
      i. Was it helpful or not? Why?
      ii. What about where the visit was held, were you comfortable, uncomfortable in the space? Was there enough privacy, why or why not? (probe around stigma)
4. Now let’s talk about behavioral health care services available in your community. What kind of services already exist in your community?
   a. Probe:
      i. Services through the church or other organizations?
      ii. Services for individuals and families that help with trauma/stress related to the COVID-19 pandemic (e.g., grief, death, job loss, child anxiety)
      iii. Have you ever used these services, why or why not?
5. In your opinion, how could this clinic better address mental health and substance use needs in your community?
6. Is there anything else you’d like to share related to your experiences with using the services available at this clinic?
