# Supplementary material for: A Community Grounded Approach to Addressing COVID-19 Health Disparities and Misinformation in Latinx and Indigenous Mexican Immigrant Communities
Source: J Community Health. 2026 Jul 25;51(6):991–1004. doi: 10.1007/s10900-026-01615-5 (PMC13748008; doi:10.1007/s10900-026-01615-5)
Supplement: Supplementary file 3 [file 10900_2026_1615_MOESM3_ESM.docx]

Supplementary File: Pretest and Posttest Survey Questions

*MISINFORMATION*

**Please indicate whether you agree or disagree with the following statements:**

|  | Agree (1) | Neither agree nor disagree (2) | Disagree (3) |
| --- | --- | --- | --- |
| 1. The coronavirus and COVID-19 are not that serious. | 1 | 2 | 3 |
| 2. Social networks such as Facebook, Instagram, Snapchat and WhatsApp are good sources of information about COVID-19. | 1 | 2 | 3 |
| 3. People can contract the virus where they are tested for COVID-19 and at vaccination sites. | 1 | 2 | 3 |
| 4. By getting the COVID-19 vaccine, people will get the virus, get sick, and possibly die. | 1 | 2 | 3 |
| 5. Upon receiving the COVID-19 vaccine, people will be sterilized. | 1 | 2 | 3 |
| 6. I will not need to wear a mask after getting vaccinated against COVID-19. | 1 | 2 | 3 |
| 7. COVID-19 vaccines don't work because you can still get COVID after vaccination. | 1 | 2 | 3 |
| 8. I already had COVID-19 and have recovered, so I don't need to get vaccinated against COVID-19. | 1 | 2 | 3 |

*MISTRUST*

**Please indicate whether you agree or disagree with the following statements:**

|  | Agree (1) | Neither agree nor disagree (2) | Disagree (3) |
| --- | --- | --- | --- |
| 1. The spread of the coronavirus is political—it was developed by the government. | 1 | 2 | 3 |
| 2. The COVID-19 vaccine is a way for the government to control people. | 1 | 2 | 3 |
| 3. The COVID-19 vaccine is a way for the government to implant a microchip to monitor behavior and location. | 1 | 2 | 3 |
| 4. The COVID-19 vaccine is a way for the government to give another deadly virus to people. | 1 | 2 | 3 |
| 5. During the COVID-19 pandemic, hospitals let people who are low-income, Latino, or non-English-speaking die. | 1 | 2 | 3 |
| 6. The Mexican government is handling the COVID-19 pandemic better than the U.S. government. | 1 | 2 | 3 |

*FEAR*

**Please indicate whether you agree or disagree with the following statements:**

|  | Agree (1) | Neither agree nor disagree (2) | Disagree (3) |
| --- | --- | --- | --- |
| 1. If I test positive for COVID-19, I will lose my job. | 1 | 2 | 3 |
| 2. If I test positive for COVID-19, I or someone close to me, will be deported. | 1 | 2 | 3 |
| 3. COVID-19 testing sites will share my information, such as my immigration status, with the government. | 1 | 2 | 3 |
| 4. COVID-19 vaccination sites will share my information, such as my immigration status, with the government. | 1 | 2 | 3 |
| 5. I've been afraid to get tested for COVID-19 because I don't have U.S. documents. | 1 | 2 | 3 |
| 6. I have not been vaccinated against COVID-19 because I do not have U.S. documents. | 1 | 2 | 3 |

*LIMITED ENGLISH PROFICIENCY*

P**lease indicate whether you agree or disagree with the following statements:**

|  | Agree (1) | Neither agree nor disagree (2) | Disagree (3) |
| --- | --- | --- | --- |
| 1. I won't be getting tested for COVID-19 because I don't speak English well. | 1 | 2 | 3 |
| 2. I won't get the COVID-19 vaccine because I don't speak English well. | 1 | 2 | 3 |
| 3. If I get COVID-19, I won't go to the hospital because I don't speak English well. | 1 | 2 | 3 |
| 4. I have not applied for help because I do not speak English and I cannot meet the requirements they ask for. | 1 | 2 | 3 |
