# Supplementary material for: A Community Grounded Approach to Addressing COVID-19 Health Disparities and Misinformation in Latinx and Indigenous Mexican Immigrant Communities
Source: J Community Health. 2026 Jul 25;51(6):991–1004. doi: 10.1007/s10900-026-01615-5 (PMC13748008; doi:10.1007/s10900-026-01615-5)
Supplement: Supplementary file 4 [file 10900_2026_1615_MOESM4_ESM.docx]

**Supplementary Table 1. Factors underlying COVID-19 testing and vaccination decisions**

**Misinformation**

1. The coronavirus and COVID-19 are not that serious.

2. Social networks such as Facebook, Instagram, Snapchat and WhatsApp are good sources of information about COVID-19.

3. People can contract the virus where they are tested for COVID-19 and at vaccination sites.

4. By getting the COVID-19 vaccine, people will get the virus, get sick, and possibly die.

5. Upon receiving the COVID-19 vaccine, people will be sterilized.

6. I will not need to wear a mask after getting vaccinated against COVID-19.

7. COVID-19 vaccines don't work because you can still get COVID after vaccination.

8. I already had COVID-19 and have recovered, so I don't need to get vaccinated against COVID-19.

**Mistrust**

1. The spread of the coronavirus is political (e.g., it was developed by the government).

2. The COVID-19 vaccine is a way for the government to control people.

3. The COVID-19 vaccine is a way for the government to implant a microchip to monitor behavior and location.

4. The COVID-19 vaccine is a way for the government to put another deadly virus on people.

5. During the COVID-19 pandemic, hospitals let people (who are low-income, Latino, and/or non-English-speaking) die.

6. The Mexican government is handling the COVID-19 pandemic better than the U.S. government.

**Fear**

1. If I test positive for COVID-19, I will lose my job.

2. If I test positive for COVID-19, I or someone close to me will be deported.

3. COVID-19 testing sites will share my information, such as my immigration status, with the government.

4. COVID-19 vaccination sites will share my information, such as my immigration status and records, with the government.

5. I've been afraid to get tested for COVID-19 because I don't have U.S. documents.

6. I have not been vaccinated against COVID-19 because I do not have U.S. documents.

**Limited English Proficiency**

1. I won't get tested for COVID-19 because I don't speak English well.

2. I won't get the COVID-19 vaccine because I don't speak English well.

3. If I get COVID-19, I won't go to the hospital because I don't speak English well.

4. I have not sought out help because I do not speak English, and I cannot fill out the paperwork they ask for.
